# Associations Between Brief Resilience Scale Scores and Ageing-Related Domains in the Lothian Birth Cohort 1936

**DOI:** 10.1037/pag0000419

**Published:** 2019-11-04

**Authors:** Adele M. Taylor, Stuart J. Ritchie, Ciara Madden, Ian J. Deary

**Affiliations:** 1 Department of Psychology, The University of Edinburgh; 2 Social, Genetic, and Developmental Psychiatry Centre, King’s College London; 3 Centre for Dementia Prevention, The University of Edinburgh; 4 Centre for Cognitive Ageing and Cognitive Epidemiology, The University of Edinburgh

**Keywords:** Brief Resilience Scale, cognitive ability, ageing, personality, wellbeing

## Abstract

It is unclear how scores on self-report resilience scales relate to key ageing-related domains in older age and if they truly measure resilience. We examined antecedents and outcomes of age-76 Brief Resilience Scale (BRS) scores in participants of the Lothian Birth Cohort 1936 (*n* = 655). We found bivariate associations between age-76 BRS scores and ageing-relevant antecedent variables measured at least 3 years earlier, from domains of cognitive ability, physical fitness, and wellbeing and, additionally, sociodemographics and personality (absolute *r*’s from .082 to .49). Biological health variables were not associated with BRS scores. Age-73 cognitive ability (largest β = 0.14), physical fitness (largest β = 0.084), and wellbeing variables (largest β = 0.26) made positive independent contributions to age-76 BRS scores in multivariate models. In a conservative model including all variables as covariates, corrected for multiple comparisons, only emotional stability (neuroticism) significantly independently contributed to BRS score (β = 0.33). An exploratory backward elimination model indicated more wellbeing and personality associates of BRS scores (βs from .087 to .32). We used latent difference score modeling to assess outcomes of BRS scores; we examined associations between age-76 BRS and change in latent factors of age-related domains between age 76 and 79. Whereas BRS scores were related cross-sectionally to levels of latent cognitive ability (*r* = .19), physical fitness (*r* = .20), and wellbeing (*r* = .60) factors, they were not related to declines in these domains. The independence of the BRS construct from established wellbeing and personality factors is unclear.

Older age is often characterized negatively due to the increase in poor health and other adverse events experienced by many older people. Mean declines in cognitive (Salthouse, 2004) and physical functions (Cooper et al., 2011) are seen in older age. Age-related decline is evident in biological and physiological processes (López-Otín, Blasco, Partridge, Serrano, & Kroemer, 2013). Mental health changes have also been reported, including greater prevalence of depression in older age (Steffens, Fisher, Langa, Potter, & Plassman, 2009). Changes in these key age-related domains have the potential to impact negatively upon an individual’s independence, health, and wellbeing (Lara et al., 2013). Yet even when health and mental function are found to be objectively poor, some older adults report high levels of “successful ageing” (Jeste et al., 2013) and happiness (Jopp & Rott, 2006). This suggests that some people may be better able to withstand age-related changes in health and functioning than others. What accounts for this variation between individuals is still to be determined, though it has recently been suggested that resilience—the ability to adapt positively to risk or adversity—might be important in withstanding the negative consequences of ageing (MacLeod, Musich, Hawkins, Alsgaard, & Wicker, 2016; Wild, Wiles, & Allen, 2013).

In what follows, we stress that it is important to keep in mind the possible difference between the construct of resilience and the name given to the scores from a scale that purports to assess resilience. Studies that have examined so-called resilience in older age have found some evidence of associations with key age-related domains. Several have focused on relationships between scores on questionnaires that purport to assess resilience, wellbeing, and other psychological constructs. In a study of older adults (mean age 77 years; *n* = 171), geographically and demographically similar to those investigated here, moderate associations were found between scores of the self-report Brief Resilience Scale (BRS; Smith et al., 2008), better mental wellbeing, *r* = .41, *p* < .001, and fewer symptoms of anxiety and depression, *r* = −.46, *p* < .001 (Harris, Brett, Starr, Deary, & McIntosh, 2016). Other studies that used scores from the Connor-Davidson Resilience Scale (CD-RISC) found higher scores to be associated with lower daily stress (*r* = −.38, *n* = 27; Ong, Bergeman, Bisconti, & Wallace, 2006); greater emotional wellbeing (*r* = .49, *n* = 1,395) and optimism (*r* = .44, *n* = 1,395; Lamond et al., 2008); and lower levels of loneliness (*r* = −.29, *n* = 2,025; Kuwert, Knaevelsrud, & Pietrzak, 2014).

In addition to psychological health, physical health has also been related to various self-report measures purporting to assess resilience. Perna et al. (2012) examined differences in health behaviors between individuals with high and low scores on a short version of the Resilience Scale (RS). They found that, in individuals aged ≥ 65 years (*n* = 3,347), higher RS scores were associated with greater participation in physical activity (odds ratios [*OR*] from 1.9 to 2.2). Higher scores on other scales intended to measure resilience have also been linked to better self-rated health (relative risk [RR] = 1.65) and greater grip strength (RR = 1.40, *n* = 546; Hardy, Concato, & Gill, 2004); reduced risk of disability in activities of daily living (*OR* = 1.04, *n* = 11,112; Yang & Wen, 2014); and increased longevity (*OR* = 1.43, *n* = 1,528; Zeng & Shen, 2010).

Other domains affected by ageing have received less attention in the literature in relation to resilience. In a review and concept analysis of resilience, Windle (2011) noted that “neuroscience/biological approaches to resilience are notably missing . . . a major contribution to resilience research could be made through more multi-disciplinary studies that examine . . . its role in healthy ageing and managing loss, such as changes in cognitive functioning” (p. 155). There is limited evidence on the relationship between resilience and cognitive ability in older age. Research based on children and adolescents suggested that individuals with higher IQ tend to have higher resilience despite experiencing adversity, as evidenced by better developmental outcomes (Masten et al., 1999). Better performance on psychometric tests of executive function and processing speed in early adulthood were associated with higher scores on the Resilience Scale for Adults (RSA; Stainton, Chisholm, Upthegrove, & Wood, 2018). Studies specific to older age are rarer, and although there is some evidence to suggest that self-reported resilience scale scores relate to subjective ratings of cognitive ability in later life, support for an association with objective measures of cognitive ability is weaker. Lamond et al. (2008) found that higher CD-RISC scores were moderately negatively correlated with subjective cognitive function in a sample of 1,395 older women aged 73 years (*r* = −.40). However, the association with an objective cognitive measure was null (*r* = .065). A smaller study of 129 individuals aged 77 found nonsignificant associations between self-reported BRS scores and objective measures of verbal ability (*r* = .13) and nonverbal reasoning (*r* = .07). Additionally, no relation was found between BRS scores in older age and individual differences in cognitive change between ages 11 and 77 years (Harris et al., 2016).

There is also limited evidence on the relationship between resilience and biological processes. Many physiological systems are implicated in an individual’s response to stress and adversity (Feder, Nestler, & Charney, 2009). Furthermore, functional declines in several physiological systems are considered to be hallmarks of ageing (López-Otín et al., 2013). However, few studies have investigated whether there is an association between resilience and physiological and biological markers of stress or ageing, and no studies have examined this association exclusively in older people. A study of diabetic patients aged between 18 and 75 years reported an interaction between a resilience factor (derived from self-reported optimism, self-esteem, self-efficacy, and self-mastery measures) and psychological distress in predicting glycaemia levels 1 year later, such that, for those with low to moderate but not high resilience, increased distress related to worsening glycaemic levels over time (β = −0.52; Yi, Vitaliano, Smith, Yi, & Weinger, 2008). In a small study examining resilience (derived from a subset of questions on the Defense Style Questionnaire) and the stress hormone cortisol in adults aged between 18 and 60, Simeon et al. (2007) found that self-reported resilience was positively correlated with urinary cortisol levels (*r* = .28). However, no association was found between RS scores and a range of psychopathology-related biological markers in either patients diagnosed with mental health conditions or healthy controls (Mizuno et al., 2016).

As has been described, much of the literature on resilience in older age is based on scores from self-report resilience scales or resilience factors derived from other scales. It is necessary to question whether they truly measure the construct of resilience. These various scales inherently treat resilience as a trait that exists on a continuum within all individuals. However, there is growing consensus that resilience is not an individual trait but should be understood as the capacity of dynamic systems to adapt to adversity. From a systems perspective, resilience changes over time and across different contexts (Masten, 2016; Southwick, Bonanno, Masten, Panter-Brick, & Yehuda, 2014) and cannot be measured directly; rather, it is inferred through examining adversity and adaptive response (Cosco et al., 2017). Therefore, the resilience label given to these scales may be a misnomer, as they potentially represent some other construct. However, if they are not assessing true resilience in this sense, then it is important to ask what the scales measure and understand their nomological networks. This important theoretical job is partnered by a practical one. So-called resilience scales are widely used and have produced many results. Therefore, it is helpful to understand what they have assessed, and part of that is by way of understanding the personal variables to which they relate.

The question of the construct validity of self-reported, so-called resilience is difficult to answer, partly due to the broad range of self-report scales and factors that have been used to represent resilience in the field. Resilience scales vary in content but are typically composed of items relating to manifest characteristics or personality traits believed to enable individuals to thrive following adversity. The 25 items of the CD-RISC relate to constructs such as hardiness, control, self-esteem, coping style, and stress resistance (Connor & Davidson, 2003). The RS consists of 25 items relating to equanimity, perseverance, self-reliance, meaningfulness, and existential aloneness (Wagnild & Young, 1993). The RSA includes social and familial components in addition to personal qualities (Friborg, Hjemdal, Rosenvinge, & Martinussen, 2003). The BRS stands apart from other scales by being the only one with items that directly address an individual’s capacity to “bounce back” rather than the traits that make this possible. So-called resilience scales might also be an example of the “jangle” fallacy, whereby scales with different names in fact largely measure the same underlying construct (Kelley, 1927, p. 62); that is, resilience scales might actually measure a well-established construct, such as an aspect of the personality trait of emotional stability (the opposite of neuroticism).

More evidence on the convergent and discriminant validity of the resilience scales would help to address these concerns. Evidence of incremental predictive validity would also be helpful in showing that there is something unique about the construct measured by resilience scales that makes them a useful measurement tool in addition to, or in place of, other already-established measures of other constructs. The current study is focused on the self-report BRS, which is reported to have good convergent validity. It has moderate to strong correlations with other self-report resilience scales (*r*’s up to .72) and moderate associations with related constructs that are in the appropriate direction; for example, it has moderate positive correlations with optimism and positive affect and moderate negative correlations with anxiety and depression (Chmitorz et al., 2018; Rodríguez-Rey, Alonso-Tapia, & Hernansaiz-Garrido, 2016; Smith, Tooley, Christopher, & Kay, 2010). Discriminant predictive validity was also reported based on significant partial correlations between BRS scores and a range of health outcomes following adjustment for optimism, Type D personality, and social support (Smith et al., 2010). However, apart from the findings on Type D personality, there has been little examination of how the BRS relates to personality traits; therefore, it is unclear if BRS scores are substantially independent of established personality factors. Moderate correlations have been reported between other resilience measures and personality traits (Oshio, Taku, Hirano, & Saeed, 2018). Of particular interest here is the relationship between BRS scores and emotional stability/neuroticism. Neuroticism (and its inverse, emotional stability) describes a tendency toward being sensitive and experiencing anxiety, depression, and other negative emotions. The BRS and some facets of neuroticism both relate to responses to stressful or upsetting circumstances. Only one study has reported on the association between neuroticism and scores on the BRS, and none specifically studied older adults. Navrady et al. (2018) found a moderate negative correlation between neuroticism and BRS score, *r* = −.48, *p* < .001, in adults of mean age 56 years. In a recent meta-analysis based on 30 studies (*N* = 15,609), the estimated average correlation between neuroticism and various resilience measures was *r* = −.46 (Oshio et al., 2018).

Direct comparison of the associates of the BRS scores and measures of neuroticism in older age are limited by the low number of studies that have examined BRS scores in this age group and the differences in methods of measuring associate variables. However, comparison of studies with samples of different ages shows a substantial overlap between the predictors and outcomes of neuroticism and BRS scores. In cross-sectional studies, lower resilience scale scores relate to more symptoms of ill health (*r*’s from −.28 to −.50; Smith et al., 2008), greater symptoms of anxiety and depression (*r* = −.46), and greater loneliness (*r* = −.23; Harris et al., 2016). Higher resilience scores have been associated with greater optimism (*r* = .44; Lamond et al., 2008). Similarly, higher emotional stability (inverse of neuroticism) has been associated with fewer symptoms of anxiety (*r* = −.31) and depression (*r* = −.53; Laukka, Dykiert, Allerhand, Starr, & Deary, 2018) and greater optimism (*r* = .49; Taylor, Ritchie, & Deary, 2017). Moreover, higher neuroticism has been linked to a greater number of medical conditions (*r* = .19; Neeleman, Bijl, & Ormel, 2004) and greater loneliness (*r* = .28; Wang & Dong, 2018). The same is found when comparing results from studies of older adults that used different resilience scales. As described earlier, older age resilience scale scores have been associated with better subjective cognitive function and better physical function, and they predict increased longevity (Hardy et al., 2004; Lamond et al., 2008; Zeng & Shen, 2010). Similarly, associates of neuroticism in older age include poorer subjective cognitive function (Slavin et al., 2010), and outcomes include declines in physical function (Buchman et al., 2013) and increased risk of mortality (Wilson, Krueger et al., 2005). There is evidence, therefore, that makes it worth asking whether self-reported resilience scale scores are, to a substantial extent, jangles of emotional stability (negative neuroticism).

The aim of the present study was to understand more about key age-related antecedents and outcomes of BRS scores in later life. Data are from the Lothian Birth Cohort 1936 (LBC1936), a community-dwelling narrow-age cohort. The BRS was first administered to LBC1936 participants at age 76 years; here, we examined associations between age-76 BRS scores and potential antecedents from a range of domains, including cognitive ability, physical fitness, biological health, and wellbeing measured at age 73. A previous study based on a geographically similar but smaller and older sample showed that later-life BRS scores were associated with childhood measures of personality and illness (Harris et al., 2016). As such, here we also used life-course data to examine whether early life and earlier adulthood sociodemographic factors were antecedents to older-age BRS scores. Furthermore, because personality traits are known to correlate moderately with resilience scale scores, and as such represent a potential source of confounding (or jangling), we examine the effect of the Big Five personality traits on these associations. We also investigate outcomes of BRS scores to find whether BRS scores had predictive capability in relation to some of the common declines of older age. We examined associations between age-76 BRS scores and the trajectory of change in the latent factors of the key age-related domains of cognitive ability, physical fitness, biological health, and wellbeing between age 76 and 79 years. For reasons already described, this study does not advocate for or against the BRS scores examined here being interpreted as a measure of true resilience; the purpose of the study was to understand more about this scale that is commonly used in the field and has previously been reported on in the literature in the context of resilience.

## Method

### Participants

**Figure 1 fig1:**
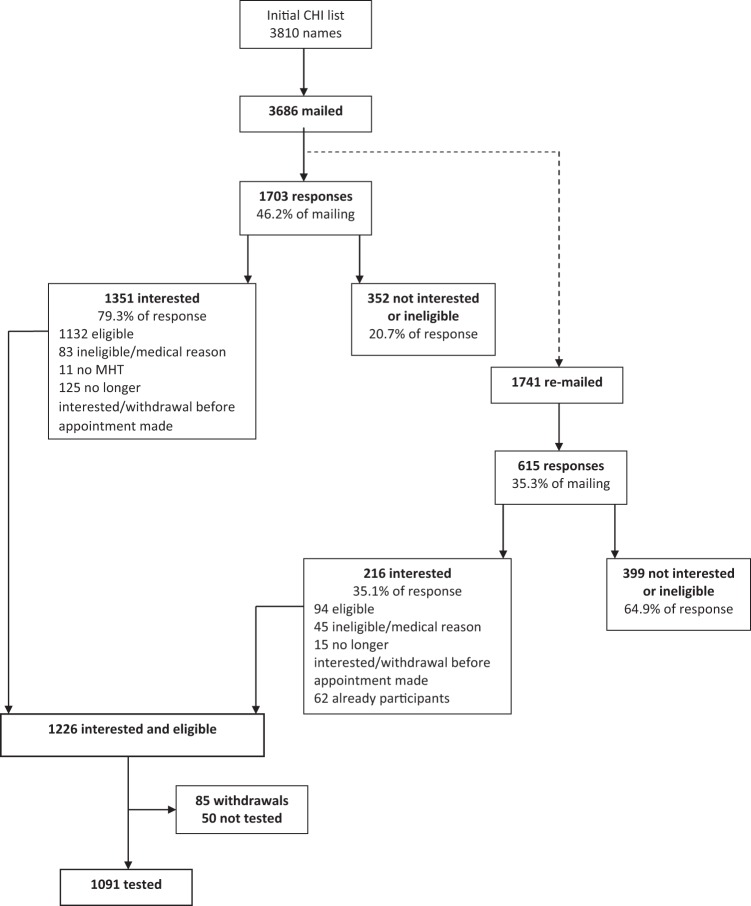
Recruitment flowchart for the Lothian Birth Cohort 1936. From “The Lothian Birth Cohort 1936: A study to examine influences on cognitive ageing from age 11 to age 70 and beyond” by I. J. Deary et al. 2007, *BMC Geriatrics, 7*, p. 5. Copyright 2007 the Authors.

Participants were members of the LBC1936, a longitudinal cohort study of cognitive, brain, and general ageing. Recruitment and assessment procedures for the study have been described comprehensively (Deary et al., 2007; Deary, Gow, Pattie, & Starr, 2012; Taylor, Pattie, & Deary, 2018). Briefly, LBC1936 participants are mostly surviving participants of the Scottish Mental Survey of 1947 (SMS1947; Scottish Council for Research in Education, 1949). In the SMS1947, 70,805 children out of a possible 75,211 children born in 1936 and attending school in Scotland on June 4th 1947 completed the Moray House Test No.12 test of general intelligence. Almost six decades later, the LBC research team identified individuals born in 1936, and thus potential surviving members of the SMS1947, currently residing in Edinburgh city and the surrounding Lothian area. Identification was carried out using the Community Health Index (CHI), which lists all individuals in a given area registered with a general medical practitioner (GP). The Lothian CHI identified 3,810 people born in 1936, and 3,686 of those individuals were invited to participate in the LBC1936 study between 2004 and 2006. Additionally, some participants were made aware of the study through media advertisements. Overall, 2,318 responses were received, and there were 1,226 interested and eligible participants (97 from media advertisements). In total, 1,091 were recruited to the LBC1936 study and were tested at Wave 1 (548 male). See Figure 1 for more details on recruitment.[Fig-anchor fig1]

Participants have since been followed-up with on three further occasions in older age: Wave 2 testing occurred between 2007 and 2010 *n* = 866; mean age = 72.5 years (*SD* = 0.71); Wave 3 testing occurred between 2011 and 2013 *n* = 697; mean age = 76.3 years (*SD* = 0.68); and Wave 4 testing occurred between 2014 and 2017 *n* = 550; mean age = 79.3 years (*SD* = 0.62). Henceforth, we will refer to these waves of testing as age 70, 73, 76, and 79, respectively. Of the 225 participants who dropped out of the study following Wave 1, 39 cases were due to death. Of the 169 participants who dropped out of the study following Wave 2, 38 cases were due to death. Of the 158 participants who dropped out of the study following Wave 3, 40 cases were due to death. Although reason for dropout is not collected systematically, from the data that are available, we know that ill health is another major cause of attrition. Note that 11 participants who did not attend Wave 3 returned for testing at Wave 4, accounting for the mismatch between Wave 3 dropout and Wave 4 attended numbers.

Ethical permission for the LBC1936 study protocol was obtained from the Multi-Centre Research Ethics Committee for Scotland (Wave 1: MREC/01/0/56), the Lothian Research Ethics Committee (Wave 1: LREC/2003/2/29), and the Scotland A Research Ethics Committee (Waves 2, 3, and 4: 07/MRE00/58). The research was carried out in compliance with the Helsinki Declaration. Written, informed consent was given by all participants.

### Measures

#### Brief resilience scale scores

BRS scores were measured for the first time in older age at age 76 years (LBC1936 Wave 3) using the BRS (Smith et al., 2008). The BRS is a short self-report psychometric measure that is described as assessing resilience in terms of the original meaning of the word: the ability to bounce back or recover from stress and adversity. The scale has six items; three are worded positively (e.g., “I tend to bounce back quickly after hard times”) and three are worded negatively (e.g., “I have a hard time making it through stressful events”). Participants responded to each item on a 5-point Likert-type scale, ranging from 1 (*strongly disagree*) to 5 (*strongly agree*). Negatively worded items were reverse scored, and the overall BRS score was the mean of the six items, with higher scores indicating greater resilience. The BRS was included in a booklet containing 15 different questionnaires that was mailed to participants prior to attending age-76 follow-up testing. Participants returned the completed booklet during their follow-up visit where it was checked by a trained researcher, and any errors in types of response, such as giving two answers to a single question, or omissions, were corrected.

#### Key ageing domains

All variables relating to the key ageing domains examined in the current study (cognitive ability, physical fitness, biological health, and wellbeing) were measured in the same way and using the same equipment during follow-up testing at ages 73, 76, and 79 years. Cognitive, physical, and biological factors were measured on the same day as each other as part of the follow-up testing visit. Wellbeing variables were completed either in the days prior to the appointment or on the same day.

##### Cognitive ability

Participants were administered a wide-ranging battery of cognitive tests by trained researchers at each follow-up wave. For the purposes of the current study, we selected the Symbol Search, Digit Symbol Substitution, Matrix Reasoning, Letter-Number Sequencing, and Block Design subtests from the Wechsler Adult Intelligence Scale, 3rd UK Edition (Wechsler, 1998a, 1998b), and the Digit Span Backwards subtest from the Wechsler Memory Scale, 3rd UK Edition (Wechsler, 1998a, 1998b). For latent difference score analysis, we derived a factor of general cognitive ability from the six subtests, as has been done in previous studies based on this cohort (e.g., Luciano et al., 2009).

##### Physical fitness

Trained research nurses measured three aspects of physical fitness during each follow-up wave. Grip strength in the right hand was recorded using a North Coast Hydraulic Hand Dynamometer. Lung function was measured as forced expiratory volume in 1s and assessed using a Micro Medical Spirometer. The current analyses used the best of three attempts on both the grip strength and lung function measures. Walk speed was recorded on a stopwatch and was the time taken (in seconds) for participants to walk 6m along a corridor. Higher walk speed scores reflect poorer (i.e., slower) performance. All three physical fitness measurements were adjusted for sex and height (measured on the day of the physical fitness assessment).

##### Biological health

We calculated three measures of biological health (allostatic load, DNA methylation age acceleration, telomere length), from blood samples and other physiological indices, that have not been previously examined in relation to resilience scale scores. Allostatic load is a hypothesized trait representing developmental build-up of stress and “wear and tear” on bodily subsystems (McEwen, 1998). The other two biological health measures have previously been proposed as “biological clocks” used to examine differences in biological age between individuals of the same chronological age (Marioni et al., 2016): telomere length and DNA methylation. Telomeres are sections of DNA and protein that act as a protective cap at the end of chromosomes. Telomere length is considered to be a marker of biological age (López-Otín et al., 2013). Telomere length decreases with age and in response to cell division and various types of damage and has been related to disease and mortality in humans (Blackburn, Epel, & Lin, 2015). DNA methylation is the chemical modification of the genome related to the regulation of genes. Due to the significant effect of chronological age on methylation levels, DNA methylation-based biological markers of ageing, so-called “epigenetic clocks,” have been developed as a measure of the difference between an individual’s chronological age and their methylation-indicated, “biological” age. Accelerated ageing as predicted by a faster-running epigenetic clock has been related to multiple health outcomes including all-cause mortality (Marioni et al., 2015).

The allostatic load variable used in the current study was calculated using the second-order multigroup confirmatory factor analytic method described by Booth, Starr, & Deary (2013; see Figure 1 of that paper). This model included measurement of 10 biomarkers identified to represent different contributing factors to allostatic load: body mass index (BMI), triglyceride, high-density (HDL) and low-density lipoprotein (LDL), glycated hemoglobin (HbA1c), fibrinogen, and mean systolic blood pressure (SBP) and mean diastolic blood pressure (DBP). The biomarkers triglyceride, HDL, LDL, HbA1c, and fibrinogen were analyzed at the Department of Laboratory Medicine, Western General Hospital, Edinburgh. BMI was calculated as weight (in kilograms) over height (meters squared). SBP and DBP were calculated as the average of three seated readings taken using an Omron 705IT blood pressure monitor. A latent construct of allostatic load was calculated using the second-order multigroup confirmatory factor analytic method.

For measurement of telomere length, DNA was extracted from whole blood by standard procedures at the WTCRF Genetics Core, Western General Hospital, Edinburgh. Telomere length was measured using a quantitative real-time polymerase chain reaction (PCR) assay at the University of Newcastle. All PCRs were carried out on an Applied Biosystems (Pleasonton, CA, U.S.) 7900HT Fast Real Time PCR machine with 384-well plate capacity. Full details are reported in Martin-Ruiz et al. (2004). Four internal control DNA samples were run within each plate to correct for plate-to-plate variation. These internal controls are cell lines of known absolute telomere length. The relative ratio values (telomere starting quantity/glyceraldehyde 3-phosphate dehydrogenase starting quantity) were used to generate a regression line by which values of relative telomere length for the actual samples were converted into absolute telomere lengths. The correlation between relative and absolute telomere lengths was 0.8.

To calculate DNA methylation age acceleration, DNA was extracted from whole blood samples and methylation typing was performed at the WTCRF Genetics Core at the Western General Hospital, Edinburgh. DNA methylation was measured at 485,512 sites using the Illumina HumanMethylation450 BeadChip array. Bisulphate-converted DNA samples were hybridized to the Infinium HumanMehtylation450 array using the Infinium HD Methylation protocol and Tecan robotics (Illumina). Raw intensity data were background-corrected and normalized using internal controls, and methylation beta values were generated using the R minfi package (Aryee et al., 2014). Quality control (QC) was carried out on these data to remove low-quality samples, probes with a low detection rate, samples with a low call rate, and samples where there was a sex mismatch based on XY probes. Post-QC, there were 450,726 autosomal probes available for analysis. Full details are reported in Shah et al. (2014). Seventy-one of these probes were used to calculate DNA methylation age using the regression weights supplied by Hannum et al. (2013). The DNA methylation-based age acceleration measure used in the current paper was calculated by regressing DNA methylation age on chronological age and saving the residual.

##### Wellbeing

Psychological wellbeing was measured with three self-report scales. As with the BRS, two of these scales were included in a booklet of questionnaires mailed to participants prior to attending their follow-up visits. Questionnaires were checked by a trained researcher on the day of follow-up testing, and any errors and omissions were corrected by the participant at this time.

Mental wellbeing was assessed using the Warwick-Edinburgh Mental Well-Being Scale (WEMWBS; Tennant et al., 2007), which was developed to capture the affective-emotional, cognitive-evaluative, and psychological functioning aspects of subjective wellbeing. Participants responded to 14 items (e.g., “I’ve been feeling confident”) on a 5-point Likert scale ranging from 1 (*none of the time*) to 5 (*all of the time*) by selecting the response that best described their experience in the previous two weeks. The overall WEMWBS score was the sum of responses to all 14 items, with higher scores indicating better mental wellbeing.

Satisfaction with life was measured using the Satisfaction with Life Scale (SWLS; Diener, Emmons, Larsen, & Griffin, 1985), a 5-item measure of global cognitive judgments of life satisfaction, developed to measure the nonemotional component of subjective wellbeing. Participants responded to items (e.g., “In most ways my life is close to ideal”) on a 7-point Likert-type scale ranging from 1 (*strongly disagree*) to 7 (*strongly agree*). The overall SWLS score is the sum of all five items, with higher scores representing greater satisfaction with life.

The Hospital Anxiety and Depression Scale (HADS; Zigmond & Snaith, 1983) was used to assess current and recent anxiety and depressive mood symptoms. Participants completed the HADS at their follow-up testing visit, the same day as the cognitive, physical, and biological tests. The scale has 14 items, seven of which address anxiety (e.g., “Worrying thoughts go through my mind”) and seven depression (e.g., “I still enjoy the things I used to enjoy”). The overall HADS score is calculated as the sum of responses to all anxiety and depression items, with higher scores reflecting increased symptom severity.

#### Sociodemographics

During an interview with a trained psychologist at Wave 1 (age 70), participants retrospectively reported on socioeconomic status (SES) and education. Participant’s and their father’s occupational SES was calculated based on principal occupation before retirement, coded according to the Office of Population Censuses Surveys 1980 (Office of Population Censuses & Surveys, 1980), and General Register Office’s Census 1951 Classification of Occupations (General Register Office, 1956) respectively. For married women, their husband’s SES was used if higher than their own. SES codes ranged from 1 (*professional*) to 5 (*unskilled labor*), such that higher scores indicate lower occupational SES. Education was measured as the participant’s self-reported number of years of formal full-time education. A childhood environmental deprivation measure based on self-reported childhood living conditions was calculated according to methods reported previously (Johnson, Corley, Starr, & Deary, 2011). This composite measure was the sum of the following standardized variables: number of people per room in the home, indoor or outdoor toilet facilities, and number of people sharing toilet facilities.

#### Personality traits

The 50-item International Personality Item Pool (IPIP; Goldberg, 2001; Gow, Whiteman, Pattie, & Deary, 2005) was used to measure the Big Five personality factors: emotional stability (the inverse of neuroticism), extraversion, agreeableness, conscientiousness, and intellect. The IPIP contains 10 items for each personality factor, to which participants responded on a 5-point Likert-type scale, indicating how well the item described them from 0 (*very inaccurate*) to 4 (*very accurate*). This was included in the multiquestionnaire booklet administered at each follow-up wave.

### Statistical Analysis

Statistical analyses were carried out using IBM SPSS Statistics v.22, MPlus v.7.3 (Muthén & Muthén, 2014), and R v.3.5.1 (R Core Team, 2013). The current study was based on participants with complete BRS data at age 76 (*n* = 679). Participants with a Mini Mental State Examination score of <24, commonly used as a cutoff indicating possible dementia, were excluded (*n* = 24), leaving a sample of 655 for analyses. Outlying data points were capped at ± 3.5 standard deviations from the mean (Symbol Search, environmental deprivation, grip strength, walk speed, lung function, WEMWBS, HADS, agreeableness, conscientiousness, telomere length, DNA methylation). Principal components analysis of the BRS items confirmed there was a single resilience component: the first unrotated principal component explained 59.7% of variance, with item loadings ranging from 0.72 to 0.82 (*M* = 0.77). Cronbach’s alpha for the BRS was 0.86. *T* tests were used to examine differences in age 73 (Wave 2) scores between those who dropped out following Wave 2 and those who returned for testing at Wave 3 and to examine differences in age 76 (Wave 3) scores between those who dropped out following Wave 3 and those who returned for testing at Wave 4. No Wave 1 data were analyzed in this report. We examined antecedents of age-76 BRS scores by testing associations with early life and earlier adulthood sociodemographics (retrospectively reported at age 70) and variables from the key ageing domains (cognitive, physical, biological, wellbeing) and personality traits measured at age 73. This included both bivariate correlations and multiple linear regression analyses. For the latter, variables were entered consecutively in a priori-decided blocks to test relative importance of each variable to BRS score and to adjust for potentially confounding variables. Six models adjusted for cognitive ability, sociodemographic, physical fitness, biological health, wellbeing, and personality variables. All models were adjusted for age and sex. Finally, we applied backward elimination to all variables in the “fully adjusted” Model 6, except age and sex covariates, to retain a parsimonious model.

**Figure 2 fig2:**
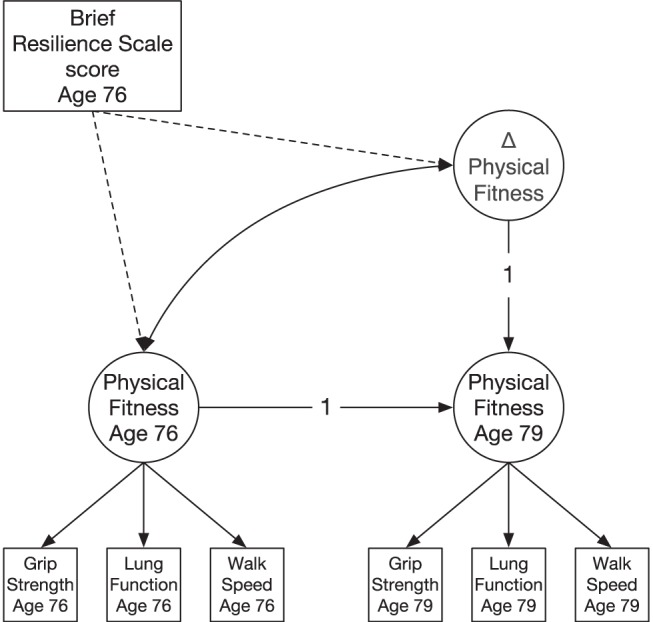
Simplified diagram of the latent difference score model. For illustration, we show the version for physical fitness: a latent factor at each of the two ages (circles) is indicated by scores on each of the three measured physical fitness variables (squares), and a latent difference score (Δ physical fitness) is derived from them. The score from the Brief Resilience Scale (rectangle) is then regressed on the baseline and the change factors (dashed lines).

To examine the outcomes of BRS scores, we tested associations between age-76 BRS scores and trajectories of change in key ageing domains. We used latent difference score models (McArdle, 2009) to test whether BRS score at age 76 was associated with changes in the latent ageing domains of cognitive ability, physical fitness, biological health, and wellbeing between ages 76 and 79. Such models rely on the existence of correlations among each of the observed variables that make up the latent factor of that construct at each of two time points; from their results, we can examine the overall degree of change in the latent construct. Also, crucially for the present study, we can add covariates (in this case BRS score) and examine the extent to which they correlate with the baseline levels of, and change in, each latent factor. Age (in days at time of testing) and sex were included as covariates in multivariate analyses. All latent constructs had strong measurement invariance imposed upon them over the two waves (see Widaman, Ferrer, & Conger, 2010). See Figure 2 for a graphical representation of the model estimated.[Fig-anchor fig2]

The number of significant tests in our analyses increased the chance of a Type I error. To minimize the potential for a false positive result, we corrected the *p* values for the results presented in Tables 1 (comparison of participant characteristics for those remaining in vs. dropped out of the study), 2 and 3 (bivariate and multiple regression associations between antecedent variables recorded at age 73 or earlier and BRS score), and 5 (latent difference score models) according to the false discovery rate (FDR) method (Benjamini & Hochberg, 1995). Results surviving FDR correction are flagged in the tables, and results both prior to and following FDR correction are described in the Results section.[Table-anchor tbl1]

## Results

Descriptive statistics for BRS scores, early life and adulthood sociodemographics, key ageing domain variables (cognitive ability, physical fitness, biological health, and wellbeing), and personality are presented in Table 1. The mean BRS score at age 76 was 3.55 (*SD* = 0.64). There was no difference in BRS score between participants who remained in the study following age-76 testing and those who dropped out of the study after this wave (*p* = .38, *d* = 0.085). At age 73, compared to those who remained in the study, participants who subsequently dropped out had significantly lower occupational SES (*p* = .043, *d* = 0.17), lower cognitive ability (*p*’s from < .001 to .041, *d*’s from 0.18 to 0.46), poorer lung function (*p* = .013, *d* = 0.22), slower walk speed (*p* < .001, *d* = 0.49), lower mental wellbeing (*p* = .003, *d* = 0.27), more anxiety and depressive symptoms (*p* = .006, *d* = 0.25), and lower conscientiousness (*p* = .035, *d* = 0.18). At age 76, compared to those who remained in the study, participants who subsequently dropped out had significantly lower occupational SES (*p* < .001, *d* = 0.34), fewer years of education (*p* = .002, *d* = 0.27), lower cognitive ability (*p*’s from <.001 to .01, *d*’s from 0.24 to 0.52), poorer lung function (*p* = .01, *d* = 0.24), slower walk speed (*p* < .001, *d* = 0.38), greater methylation age acceleration (*p* = .005, *d* = 0.27), lower mental wellbeing (*p* = .022, *d* = 0.21), and less satisfaction with life (*p* = .026, *d* = 0.21). Most of these differences remained significant following correction for multiple comparisons. Only the following differences between participants (which were close to the *p* < .05 threshold for statistical significance) did not survive FDR correction: participant social class, digit span backward, and conscientiousness at age 73 and mental wellbeing, satisfaction with life, and intellect at age 76.

### Bivariate Analyses Between Life History Variables and Ageing-Related Domains at Age 73 and Brief Resilience Scale Scores at Age 76

The majority of sociodemographic, key ageing domain, and personality variables measured at age 73 or earlier were correlated significantly with BRS score at age 76 (see Table 2). The strongest bivariate associations were between BRS score and wellbeing and personality variables (maximum *r* = .49). Better mental wellbeing (*r* = .41), greater satisfaction with life (*r* = .29), fewer anxiety and depression symptoms (*r* = −.41), higher emotional stability (*r* = .49), and higher extraversion, conscientiousness, agreeableness, and intellect (*r*’s from .17 to .27) were all significantly associated with higher BRS score. Higher BRS scores were associated with higher cognitive ability (*r*’s from .089 to .18), more years in formal education (*r* = .10), higher occupational SES (*r* = −0.082), greater grip strength (*r* = .088), and faster walk speed (*r* = −.13). Being female was associated with lower BRS score (*r* = −.084). Only the association with participant social class did not survive FDR correction. BRS score at age 76 was not significantly associated with father’s occupational SES, childhood environmental deprivation, age-73 lung function, nor any of the age-73 biological health variables (allostatic load, telomere length, methylation age acceleration; *r*’s from .012 to .044).[Table-anchor tbl2]

### Regression Analyses With Life History Variables and Ageing-Related Domains at Age 73 as Predictors and Brief Resilience Scale Scores at Age 76 as the Outcome

Table 3 gives results of hierarchical linear regression models examining associations between sociodemographics, key domains of ageing (cognitive ability, physical fitness, biological health, and wellbeing), and personality measured at age 73 or earlier and BRS score at age 76. To test the relative importance of each variable and reduce the potential for confounding by other measured variables, all variables from the bivariate analyses were entered consecutively in a priori-decided blocks. All models were adjusted for age and sex. Model 1 included the six cognitive ability variables (Symbol Search, Digit Symbol Substitution, Matrix Reasoning, Letter-Number Sequencing, Digit Span Backwards, Block Design). Sociodemographic variables (father’s and participant’s SES, education, and childhood environmental deprivation) were added in Model 2. Models 3 and 4 further included physical fitness (grip strength, lung function, walk speed) and biological health variables (allostatic load, telomere length, methylation age acceleration), respectively. Wellbeing variables (mental wellbeing, satisfaction with life, anxiety and depression symptoms) were added to Model 5. In Model 6 we adjusted for the Big Five personality factors (emotional stability, extraversion, conscientiousness, agreeableness, intellect). As a final, exploratory step of the regression analysis we applied backward elimination to the “fully adjusted” Model 6, retaining only age and sex covariates, and variables which significantly independently contributed to BRS score (*p* < .05).[Table-anchor tbl3]

**Figure 3 fig3:**
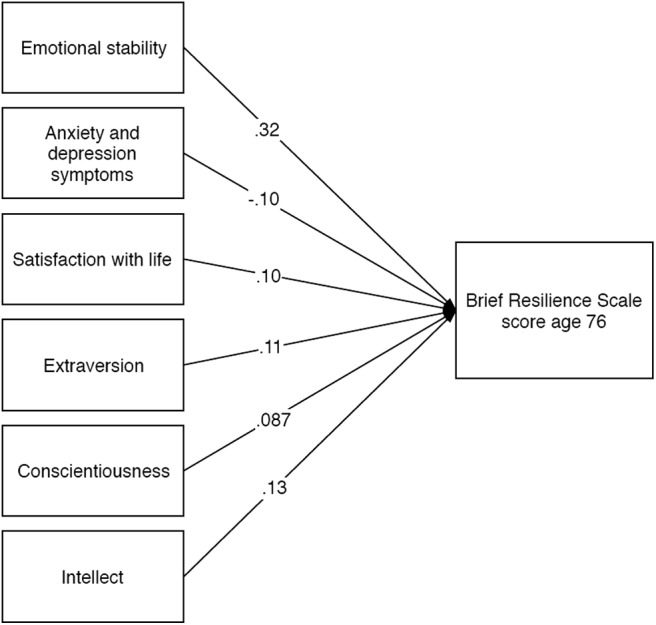
Standardized beta coefficients for linear regression (backward elimination) with ageing-related domains at age 73 as predictors and Brief Resilience Scale sores at age 76 as the outcome.

There were small positive associations between BRS score and two cognitive ability measures: block design (β = 0.14, *p* = .013) and digit symbol substitution scores (β = 0.12, *p* = .05) measured at age 73 were positively associated with age-76 BRS score. Sociodemographic variables were added in Model 2. Though sex, education, and participant social class had significant bivariate associations with BRS score, no sociodemographic variables (nor age and sex) were found to relate to BRS score in any of the multivariate models. After further adjustment for physical fitness and biological health variables in Models 3 and 4, the associations with Digit Symbol Substitution and Block Design were only slightly attenuated, though the association with Digit Symbol Substitution was no longer significant (β = 0.11, *p* = .071). There was also a small positive association between BRS score and grip strength (β = 0.078, *p* = .089) and a negative association with walk speed (β = −0.084, *p* = .076), both nonsignificant. Models 1 to 4 explained a maximum of 2.7% of the variance in age-76 BRS score. The addition of wellbeing variables in Model 5 explained a further 18% of the variance, and all three variables were significantly associated with BRS score: greater mental wellbeing (β = 0.26, *p* < .001) and satisfaction with life (β = 0.093, *p* = .042) were associated with higher BRS scores, and higher anxiety and depression symptom severity was associated with lower BRS score (β = −0.18, *p* < .001). Model 6 made final adjustments for personality factors, which accounted for an additional 7.5% of variance in BRS score. In this “fully adjusted” model, which accounted for 27% of the variance in BRS score, the strongest association was between age-73 emotional stability and BRS score (β = 0.33, *p* < .001). Other independent contributions to BRS score were made by extraversion (β = 0.10, *p* = .024) and satisfaction with life (β = 0.093, *p* = .034), but these did not survive FDR correction for multiple comparisons. When backward elimination was applied to Model 6, the variables retained were satisfaction with life (β = 0.10, *p* = .005), anxiety and depression symptoms (β = −0.10, *p* = .024), emotional stability (β = 0.32, *p* < .001), extraversion (β = 0.11, *p* = .003), conscientiousness (β = 0.087, *p* = .012), and intellect (β = 0.13, *p* = .001; see Figure 3). Together the variables explained 30.6% of the variance in age-76 BRS score and all survived FDR correction.[Fig-anchor fig3]

Taken together, the results of the regression analyses can be summarised as follows. Years of formal education reported at age 70, and cognitive ability, wellbeing, and personality variables measured prospectively at age 73 had significant associations with BRS score at age 76 such that individuals with higher cognitive ability, better mental wellbeing and satisfaction with life, and fewer anxiety and depression symptoms had higher BRS scores. However, following adjustment for other covariates and personality in multivariate analysis, and following correction for multiple comparisons, only wellbeing and personality variables made significant independent contributions to BRS score.

### Latent Difference Score Models of Brief Resilience Scale Scores at Age 76 and Change in Ageing-Related Domains From 76 to 79

The final step of our analyses used latent difference score models to examine the relationship between age-76 BRS score and concurrent (baseline) levels of latent factors of key ageing domains (cognitive ability, physical fitness, biological health, and wellbeing) and between age-76 BRS score and trajectories of change in the latent factors between age 76 and 79. First, we examined correlations among each of the observed variables hypothesized to make up the latent factor of each construct at age 76 and age 79 (see Supplementary Tables 1 and 2). Next, we examined whether there was change over time in the latent factors and the overall degree of this change. As a final step, to investigate associations between BRS score and baseline (age 76) levels of each latent construct and between BRS score and trajectory of change in each latent construct (age 76 to age 79), we added BRS score to the models as a covariate.

Examination of the correlations among the observed variables for each hypothesized latent ageing factor indicated that there were moderate correlations among the cognitive ability variables (*r*’s from .28 to .62), physical fitness variables (absolute *r*’s from .21 to .27), and wellbeing variables (absolute *r*’s from .44 to .56) at age 76 (baseline) and age 79. As such, we produced separate latent variable models using the cognitive, physical fitness, and wellbeing variables. At ages 76 and 79, the intercepts of the observed variables loaded on the respective latent factors as follows: general cognitive ability (*r* = .61 to *r* = .70); physical fitness (*r* = .43 to *r* = .53); wellbeing (*r* = .61 and *r* = .83). All loadings were significant at *p* < .001. Examination of the observed biological health variables showed that there were only small associations between them that were not consistent across baseline and age-79 measurement timepoints (maximum *r* = .14 for telomere length and methylation age acceleration at age 76 only) and were not consistently significant. As would be expected from this pattern of relations, the latent variable model was a poor fit to the data; thus, we did not produce a latent difference score model for biological health.

Table 2 shows correlations between age-76 BRS score and observed variables from each ageing domain measured at age 76 and 79. BRS score was significantly positively correlated with cognitive ability (*r*’s from .082 to .16), physical fitness (absolute *r*’s from .031 to .13), and wellbeing variables (absolute *r*’s from .30 to .47) at age 76, with only the association between BRS score and lung function being nonsignificant. BRS score was also significantly and positively correlated with cognitive ability variables (*r*’s from .057 to .12) and wellbeing variables (absolute *r*’s from .39 to .46) measured at age 79. Associations with symbol search (*r* = .057), letter-number sequencing (*r* = .073), and physical fitness variables (absolute *r*’s from .02 to .09) were of similar magnitude to those at baseline but were not significant.

Next, we used the latent difference score models to test whether each of the key ageing domains showed significant change in older age (note that these raw change estimates came from models that were not adjusted for age at testing or sex, whereas the estimates below are age- and sex-adjusted). There was significant decline across the three years in the general factors of cognitive ability (−0.311 *SD*s, *z* = −12.31, *p* < .001) and physical fitness (−0.216 *SD*s, *z* = −9.05, *p* < .001) between age 76 and 79. Wellbeing did not show significant change (0.03 *SD*s, *z* = 0.96, *p* = .34); therefore, we did not examine the association between BRS score and change in wellbeing in the final stage of this analysis.

In order to address whether individual differences in BRS score are related to baseline levels and subsequent change in key domains of ageing, we used age-76 BRS score to separately predict the intercepts (baseline) of the general factors of cognitive ability, physical fitness, and wellbeing and to predict difference scores (change) in the general factors of cognitive ability and physical fitness (that is, we included directed paths from the BRS score to the intercepts and difference scores). All models had adequate fit as indicated by the following absolute fit indices: root mean square error of approximation, comparative fit index, Tucker-Lewis index, and standardized root mean square residual (see Table 4). At baseline, BRS score was significantly and positively related to levels of general cognitive ability (*r* = .19), physical fitness (*r* = .20), and wellbeing (*r* = .60), with all *p* values <.001. These significant associations survived FDR correction. However, there were no significant associations between baseline level of BRS score and change in either cognitive ability or physical fitness (see Table 5). The association with the largest effect size was that between BRS score and change in physical fitness between age 76 and 79 (*r* = −.26), but this did not reach statistical significance (*p* = .074). The association with change in cognitive ability was minimal, *r* = .019, *p* = .86.[Table-anchor tbl4][Table-anchor tbl5]

## Discussion

In a relatively large, well-characterized, narrow-age sample of community-dwelling older adults from the LBC1936, we aimed to investigate antecedent factors associated with age-76 scores on the BRS from a wide range of early- and later-life sociodemographic variables and age-73 cognitive ability, physical fitness, biological health, wellbeing, and personality variables. We also examined whether BRS score at age 76 related to baseline levels and trajectories of change in latent factors of general cognitive ability, physical fitness, biological health, and wellbeing between ages 76 and 79. Our results make substantial contributions to understanding the nomological network (Cronbach & Meehl, 1955) for the construct measured by the BRS in older age. We found significant bivariate associations between age-76 BRS score and antecedent variables from most domains investigated, including personality traits (especially emotional stability), education, and age-73 cognitive ability, physical fitness, and wellbeing. When backward elimination was applied to our multivariate linear regression model, we found that age-73 satisfaction with life, anxiety and depression symptoms, emotional stability, extraversion, conscientiousness, and intellect, but no other ageing-related variables, made significant independent contributions to BRS scores. Though age-76 BRS scores had significant contemporaneous relationships with baseline levels of the latent factors of general cognitive ability, physical fitness, and wellbeing, we found no evidence that declines in key domains of ageing between age 76 and 79 are meaningfully (or statistically significantly) outcomes of BRS scores.

Despite there being a growing literature on the associates of self-report resilience scales’ scores in older age, the relationship between these scales and some key age-related domains, including cognitive function and biological factors, remained underexplored. This study reported findings from a range of key domains known to be affected by ageing. Importantly, due to the breadth of phenotypic data collected in the LBC1936 sample, we were able to avoid problems relating to unexamined confounders that pose a risk to studies which only examine a small number of predictors. In addition to bivariate analyses, we conducted multivariate analyses in which the same set of variables were entered into regression models in consecutive blocks, thus reducing the potential for our results to be an artifact of intercorrelations between other factors and giving a clearer indication of the variables that independently relate to older age BRS scores.

Higher scores on six neuropsychological tests covering a range of cognitive abilities were significantly associated with higher BRS scores, and the associations between BRS score and digit symbol substitution and block design (tapping information processing speed and visuospatial reasoning abilities, respectively) survived adjustment for sociodemographic, physical fitness, and biological health variables included as covariates in linear regression models. Cognitive variables, adjusted for age in days at time of testing and sex, accounted for 2.5% of the variance in age-76 BRS score. There is little prior evidence on the relationship between cognitive ability and resilience scale scores in older age. Results from two previous studies of older adults were equivocal, but our results are consistent with the direction of associations reported in those studies, which found small and mostly nonsignificant cross-sectional associations between higher BRS scores and better vocabulary and nonverbal reasoning (Harris et al., 2016) and between higher CD-RISC scores and higher mental status scores (Lamond et al., 2008). Our results also correspond with those of Stainton et al. (2018), who found in a sample of younger adults that performance on executive function and processing speed tests accounted for 4% of the variance in RSA scores. Additionally, we found a small but highly significant association between baseline levels of a latent general cognitive ability factor and BRS score (*r* = .19; *p* < .001), though there was no evidence that BRS score related to change in this latent factor within older age, despite there being significant cognitive decline between age 76 and 79 years. This extends the results of Harris et al. (2016), who found that BRS score was not related to the trajectory of change in cognitive ability across the life course between age 11 and 77.

By examining a comprehensive range of cognitive tests and including a latent-variable approach, our results provide the most robust evidence on a relationship between cognitive ability and resilience scale scores in older age to date. Methodological differences limit the comparison of results between studies, but our results add to the evidence for higher cognitive ability being linked to higher levels of the construct measured by self-report resilience scales. The BRS was designed to capture individual differences in the ability to bounce back from adverse events. The relationship between higher cognitive ability and higher BRS scores might be due to those with higher cognitive ability being better able to employ the analytic and problem-solving skills and coping mechanisms necessary to manage difficult life circumstances. However, it is important to note that, when adjustment was made for wellbeing factors in our multivariate regression analysis, the associations between cognitive functions and BRS scores were attenuated, and they were entirely diminished by adjustment for Big Five personality factors. Whereas it is unclear if a single variable or a collection of wellbeing and personality variables explains the association between BRS score and cognitive ability factors, there is evidence from previous studies that neuroticism is associated with lower cognitive function and steeper cognitive decline in older age (Wilson, Bennett, et al., 2005).

Null findings on the association between BRS scores and biological health measures in older age are novel. No studies have reported on resilience scale scores and biological health exclusively in older people, which is surprising given that resilience scores have been related to physical health symptoms and mortality (Smith et al., 2008; Zeng & Shen, 2010) and that declining physiological integrity in older age is known to represent a risk factor for illness and mortality (López-Otín et al., 2013). None of the three biological markers investigated in the current study (allostatic load, telomere length, and DNA methylation) were found to correlate with BRS score, indicating that this measure is not related to biological health. Because there were no intercorrelations between the biological health variables, we were unable to derive a reliable biological health factor to include in our latent change score modeling. As such, we are unable to comment on whether resilience is prospectively linked to changes in general biological health. It should be noted that the biological measures used in the current study represent only a very limited selection of many possible measures related to physiological stress and ageing.

The bivariate association reported between higher age-76 BRS scores and higher grip strength corroborates the findings of Hardy et al. (2004) and extends them by showing that faster walk speed is also associated with higher BRS scores. Though BRS scores did not relate to lung function in this study, baseline scores of a latent physical fitness factor derived from grip strength, walk speed, and lung function was significantly positively related to BRS score, with a similar magnitude to the association found with cognitive ability (*r* = .20). As with cognitive ability, BRS score was not related to prospective decline in physical fitness between age 76 and 79, despite there being significant decline recorded. There are multiple psychological and biological advantages of being physically fit that could potentially account for the association between physical fitness and BRS scores (see review by Silverman & Deuster, 2014). Furthermore, it is possible that subjective ratings of fitness inform the way individuals respond to the BRS. That is, individuals may take their physical status into consideration when completing the BRS, leading those who feel physically fit relative to their peers to conclude that their fitness is evidence of a good capacity to bounce back, while those who feel less physically fit might be more likely to respond negatively to the BRS.

Some of the strongest and most consistently reported associations in the literature are on the wellbeing and personality correlates of resilience scale scores. Our results corroborate those of previous studies that reported moderate associations between higher resilience scale scores, fewer anxiety and depression symptoms, and greater emotional wellbeing (Harris et al., 2016; Ong et al., 2006). We also found a positive association between greater satisfaction with life and higher BRS score and between higher scores on all the Big Five factors of personality and higher BRS score. These were some of the largest associations found in our bivariate analyses. There were *r*-values of up to .49 with emotional stability from the IPIP questionnaire. Moreover, the latent wellbeing variable derived from emotional wellbeing, satisfaction with life, and anxiety and depression symptom variables had the largest association with BRS at baseline (*r* = .60). Notably, the inclusion of wellbeing and personality variables in the fully adjusted multivariate regression model markedly attenuated the associations between BRS score and cognitive ability and physical fitness variables. This suggests that the variance captured by the BRS is most substantially related to wellbeing and personality traits (especially emotional stability/neuroticism), rather than to cognitive or physical status. Though the results of the fully adjusted model may be overly conservative, a more exploratory analysis applying backward elimination to the fully adjusted model supports this conclusion by showing that, when nonsignificant predictors are removed from the regression model, satisfaction with life, anxiety and depression symptoms, and the personality variables emotional stability, extraversion, conscientiousness, and intellect alone contribute to BRS score. This is in agreement with a study examining the convergent and discriminant validity of the RSA in relation to the Big Five personality factors and intelligence by Friborg, Barlaug, Martinussen, Rosenvinge, and Hjemdal (2005). They concluded that there was a moderate degree of redundancy between RSA scores and personality, and due to the significant loading of RSA factors onto the latent personality factors in principal component analysis, the RSA factors should not be viewed as independent of personality, but as variants of traits from the Big Five model of personality.

The close relationship between resilience scores and other positive psychological and personality factors reported by several studies, and strongly found in the present study, has led to some criticism regarding the lack of clarity on what distinguishes this construct from other similar constructs. The substantial overlap in scale content between resilience and personality scales—one facet of neuroticism on the NEO Personality Inventory relates to vulnerability to stress, and neuroticism items on the IPIP Big Five inventory also relate to stress—raises the possibility that this is an example of the jangle fallacy (Kelley, 1927). Emotional stability (the inverse of which is neuroticism) was the only variable significantly independently associated with BRS score in our regression model after adjustment for other covariates and multiple comparisons, indicating that BRS scores are substantially accounted for by individual differences in this variable; however, as previously noted, less conservative results from our backward elimination model suggest that differences in satisfaction with life, anxiety and depression symptoms, emotional stability, extraversion, conscientiousness, and intellect are also captured by the BRS. The maximum variance in BRS score explained by any regression model was 30.6%, and future studies will be required to determine how much of this residual variance is unique to a construct labeled as resilience and how much is accounted for by other facets of personality and wellbeing not measured in the current study. Though we intended to assess antecedents to BRS scores by examining associations between BRS score at age 76 and antecedent variables measured at least three years prior, the overlap in the constructs means that it is not possible in the current study to say whether personality and wellbeing factors are true antecedents to the construct captured by BRS scores or whether the BRS is, to a substantial extent, a variant of these traits.

This study has some limitations. The degree of change that could be modeled in the key domains of ageing was limited by there being only one further wave of data available, which was collected 3 years after age-76 BRS scores. This might contribute to the null findings regarding associations between BRS score and change in the cognitive ability and physical fitness factors. Furthermore, because BRS scores were measured for the first time at the third out of four waves of available data, it precluded us from examining how within-person change in BRS scores relates to changes in key ageing domains over longer periods of time. Future waves of data collection in the LBC1936 will allow us to better track within-person change in both BRS score and key ageing domains. It should also be noted that the LBC1936 are a self-selecting sample of community-dwelling older adults who are likely to be cognitively and physically fitter than the general population. As such, it is possible that participants who have experienced the greatest physical, cognitive, biological, and wellbeing declines in older age have been lost to attrition. However, importantly, because LBC1936 participants are surviving participants of the SMS1947, which assessed the cognitive ability of almost an entire population at age 11, access to historical data at population level makes it possible to understand the selected nature of the LBC1936 and to take into account restriction of range for some variables.

## Conclusion

The present study examined antecedents and outcomes of BRS scores in older age. We found that a range of antecedent age-related variables, including greater cognitive ability, physical fitness, and wellbeing at age 73 were associated with having a higher age-76 BRS score. Having more years of formal full-time education, a more professional SES, and higher levels of all Big Five personality variables also related to higher BRS scores at age 76. However, of all variables investigated, few made independent contributions to BRS score. Our most conservative linear regression model that simultaneously included all variables found the personality trait of emotional stability to be the sole independent contributor to BRS score after adjustment for multiple comparisons; however, in a backward elimination model, satisfaction with life, anxiety and depression symptoms, extraversion, conscientiousness, and intellect were also independent predictors of BRS score. Latent difference score models showed robust cross-sectional associations between BRS scores and latent general cognitive ability, physical fitness, and wellbeing factors when measured contemporaneously at baseline, but no evidence was found for associations between BRS score and future change in these key ageing domains. Our findings make novel contributions to the nomological network of the construct measured by the BRS, but we found little evidence to suggest that this construct is fully independent of other personality and wellbeing constructs.

## Supplementary Material

10.1037/pag0000419.supp

## Figures and Tables

**Table 1 tbl1:** Characteristics of the Lothian Birth Cohort 1936 Participants by Wave of Follow-Up Testing and Comparison Between Completers and Dropouts

Participant characteristic	Wave 2 (age 73)	Completer vs dropout (*p*^a^)	Cohen’s *d*	Wave 3 (age 76)	Completer vs dropout (*p*^b^)	Cohen’s *d*	Wave 4 (age 79)
*N* (completer/dropout)	866	697/169		697	550/158^c^		550
Age (years)	72.5 (.71)	.10	.14	76.2 (.67)	.62	.054	79.3 (.63)
Sex (male)	51.1%	—	—	51.1%	—	—	49.6%
Brief Resilience Scale score	—	—	—	3.55 (.64)	.38	.085	—
Socio-demographics							
Father’s social class^d^	2.91 (.95)	.16	.13	2.89 (.95)	.56	.054	2.88 (.96)
Participant’s social class^d^	2.25 (.83)	.043	.17	2.23 (.84)	<.001^†^	.34	2.17 (.83)
Education	10.8 (1.14)	.26	.10	10.8 (1.14)	.002^†^	.27	10.9 (1.18)
Childhood environmental deprivation	−.12 (2.22)	.14	.13	−.18 (2.21)	.65	.043	−.19 (2.27)
Cognitive ability							
Symbol Search	24.8 (6.07)	<.001^†^	.37	24.9 (6.25)	<.001^†^	.44	23.0 (6.40)
Digit Symbol Substitution	56.8 (12.2)	<.001^†^	.46	54.3 (12.7)	<.001^†^	.52	51.9 (12.5)
Matrix Reasoning	13.3 (4.95)	<.001^†^	.40	13.2 (4.85)	.010^†^	.24	13.1 (5.00)
Letter-Number Sequencing	11.0 (3.05)	<.001^†^	.43	10.6 (2.95)	<.001^†^	.33	10.2 (2.79)
Digit Span Backwards	7.86 (2.28)	.041	.18	7.84 (2.36)	.005^†^	.27	7.63 (2.14)
Block Design	33.9 (10.1)	<.001^†^	.36	32.6 (9.79)	.008^†^	.24	31.6 (9.36)
Physical fitness							
Grip strength^e^	28.7 (9.41)	.063	.16	27.7 (9.76)	.45	.069	26.2 (9.45)
Lung function^f^	2.30 (.68)	.013	.22	2.11 (.62)	.011^†^	.24	2.11 (.64)
Walk speed^g^	4.30 (1.11)	<.001^†^	.49	4.65 (1.40)	<.001^†^	.38	5.14 (1.56)
Biological health							
Allostatic load	0 (1.53)	.86	.015	0 (1.45)	.31	.10	0 (1.54)
Telomere length	3958 (688)	.95	.005	3740 (668)	.32	.090	3619 (566)
Methylation age acceleration	−1.53 (6.88)	.89	.012	.62 (6.82)	.005^†^	.27	3.16 (6.20)
Wellbeing							
WEMWBS	51.5 (7.42)	.003^†^	.27	51.6 (7.88)	.022	.21	51.8 (8.27)
SWLS	25.5 (5.93)	.053	.17	25.7 (5.92)	.026	.21	25.9 (5.80)
HADS	7.07 (4.38)	.006^†^	.25	7.50 (4.45)	.077	.16	7.08 (4.45)
Personality							
IPIP emotional stability	25.0 (7.76)	.061	.16	25.3 (7.10)	.082	.16	25.4 (7.49)
IPIP extraversion	21.6 (7.28)	1.00	<.001	21.7 (7.21)	.47	.069	21.4 (7.49)
IPIP conscientiousness	27.8 (6.09)	.035	.18	28.0 (6.09)	.80	.024	27.6 (6.13)
IPIP agreeableness	31.0 (5.45)	.36	.08	30.8 (5.39)	.35	.088	30.9 (5.46)
IPIP intellect	23.8 (5.95)	.23	.11	24.0 (5.75)	.046	.19	24.1 (5.97)
*Note*. Values given are mean and standard deviation. WEMWBS = Warick-Edinburgh Mental Wellbeing Scale; SWLS = Satisfaction with Life Scale; HADS = Hospital Anxiety and Depression Scale; IPIP = International Personality Item Pool. ^†^ Results significant at *p* < .05 after false discovery rate adjustment.							
^a^ *p*-value for differences in Wave 2 (age 73) scores between those who dropped out following Wave 2 and those who returned for testing at Wave 3. ^b^ *p*-value for differences in Wave 3 (age 76) scores between those who dropped out following Wave 3 and those who returned for testing at Wave 4. ^c^ The mismatch between Wave 3 dropout and Wave 4 attended numbers is due to 11 participants who did not attend Wave 3, who did not drop out, returning to participate in Wave 4. ^d^ Social class (occupational) was categorised from 1 (*professional*) to 5 (*unskilled*) labour. ^e^ Grip strength reported as best of three attempts from right hand (kg). ^f^ Lung function was forced expiratory volume in 1 s. ^g^ Walk speed was time to walk 6 m in seconds.							

**Table 2 tbl2:** Correlations Between Early-Life and Antecedent Variables Measured at Age 73 and Brief Resilience Scale (BRS) Score at Age 76 in LBC1936 and Between BRS Score at Age 76 and Key Ageing Domain Variables Measured at Ages 76 and 79

Antecedent variables	*r* (age 73)	*r* (age 76)	*r* (age 79)
Age (in days)	−.001		
Sex	−.084*^†^		
Sociodemographics^a^			
Father’s social class^b^	−.021		
Participant’s social class^b^	−.082*		
Education^c^	.10**^†^		
Childhood environmental deprivation	−.043		
Cognitive ability			
Symbol Search	.089*^†^	.16**^†^	.057
Digit Symbol Substitution	.14**^†^	.15**^†^	.11*^†^
Matrix Reasoning	.092*^†^	.16**^†^	.12**^†^
Letter-Number Sequencing	.13**^†^	.090*^†^	.073
Digit Span Backwards	.12**^†^	.082*	.11*^†^
Block Design	.18**^†^	.091*	.12**^†^
Physical fitness			
Grip strength^d^	.088*^†^	.13**^†^	.086
Lung function^e^	.018	.031	−.020
Walk speed^f^	−.13**^†^	−.11**^†^	−.060
Biological health			
Allostatic load	.012	−.003	−.007
Telomere length	−.019	−.001	.002
Methylation age acceleration	.044	.027	.065
Wellbeing			
WEMWBS	.41**^†^	.47**^†^	.44**^†^
SWLS	.29**^†^	.30**^†^	.39**^†^
HADS	−.41**^†^	−.46**^†^	−.46**^†^
Personality			
IPIP emotional stability	.49**^†^		
IPIP extraversion	.27**^†^		
IPIP conscientiousness	.24**^†^		
IPIP agreeableness	.17**^†^		
IPIP intellect	.24**^†^		
*Note*. Coding for binary variables was as follows: male = 0, female = 1. WEMWBS = Warwick-Edinburgh Mental Wellbeing Scale; SWLS = Satisfaction with Life Scale; HADS = Hospital Anxiety and Depression Scale; IPIP = International Personality Item Pool.			
^a^ Sociodemographics were retrospectively reported at LBC1936 Wave 1 (mean age 70 years), as described in Method. ^b^ Social class (occupational) was categorised from 1 (*professional*) to 5 (*unskilled*) labour. ^c^ Education was number years in formal full-time education. ^d^ Grip strength was reported as best of three attempts from right hand (kg). ^e^ Lung function was forced expiratory volume in 1 s. ^f^ Walk speed was time to walk 6 m in seconds.			
^†^ Results significant at *p* < .05 after false discovery rate adjustment. * *p* < .05. ** *p* < .01.			

**Table 3 tbl3:** Multiple Linear Regression Results (Standardised Betas) for Antecedents of Age 76 Brief Resilience Scale Score

Independent variables	Model 1	Model 2	Model 3	Model 4	Model 5	Model 6
Age	.016	.015	.028	.029	.030	.027
Sex	−.061	−.059	−.065	−.058	−.066	−.047
Cognitive ability						
Symbol Search	−.077	−.076	−.079	−.077	−.039	−.037
Digit Symbol Substitution	.12*	.12*	.11	.11	.080	.060
Matrix Reasoning	−.051	−.048	−.058	−.056	−.035	−.034
Letter-Number Sequencing	.036	.039	.040	.041	.018	.023
Digit Span Backwards	.063	.065	.055	.056	.057	.047
Block Design	.14*	.14*	.13*	.12*	.045	.058
Socio-demographics						
Father’s social class^a^		<.001	<.001	−.005	−.032	−.014
Participant’s social class^a^		.003	.002	<.001	−.009	.020
Education^b^		−.037	−.045	−.042	−.022	−.004
Childhood environmental deprivation		−.019	−.023	−.023	.035	.036
Physical ability						
Grip strength^c^			.076	.078	.053	.042
Lung function^d^			−.018	−.013	−.045	−.043
Walk speed^e^			−.083	−.084	−.039	−.043
Biological health						
Allostatic load				.032	−.009	.018
Telomere length				.007	.003	.015
Methylation age acceleration				.030	.031	.026
Wellbeing						
WEMWBS					.26**	.080
SWLS					.093*	.093*
HADS					−.18**	−.024
Personality						
IPIP emotional stability						.33**^†^
IPIP extraversion						.10*
IPIP conscientiousness						.075
IPIP agreeableness						.008
IPIP intellect						.054
Adjusted R^2^	.025	.019	.027	.023	.20	.27
Δ R^2^	.038*	.001	.013	.002	.18*	.075*
*Note*. *N* = 522. Coding for binary variables was as follows: male = 0, female = 1. Data are presented as standardised beta coefficients reflecting change in age 76 resilience score associated with an increase of 1 *SD* unit in predictor variable. WEMWBS = Warwick-Edinburgh Mental Wellbeing Scale; SWLS = Satisfaction with Life Scale; HADS = Hospital Anxiety and Depression Scale; IPIP = International Personality Item Pool.						
^a^ Social class (occupational) was categorised from 1 (*professional*) to 5 (*unskilled*) labour. ^b^ Education was number years in formal full-time education. ^c^ Grip strength was reported as best of three attempts from right hand (kg). ^d^ Lung function was forced expiratory volume in 1 s. ^e^ Walk speed was time to walk 6 m in seconds.						
^†^ Results significant at *p* < .05 after false discovery rate adjustment (FDR adjustment applied to Model 6 results only). * *p* < .05. ** *p* < .01.						

**Table 4 tbl4:** Model Fit Indices of Latent Difference Score Models Predicting Change in Key Ageing Domains From Brief Resilience Scale Score

Model	χ^2^	*df*	*p*	RMSEA	CFI	TLI	SRMR
General cognitive ability	220.39	46	<.001	.076	.94	.93	.059
Physical fitness	12.71	14	.55	.000	1.00	1.00	.023
Wellbeing	37.58	14	.001	.051	.99	.98	.032
*Note*. RMSEA = root mean square error of approximation; CFI = comparative fit index; TLI = Tucker-Lewis index; SRMR = standardized root mean square residual.							

**Table 5 tbl5:** Associations of Age 76 Brief Resilience Scale (BRS) Score With Key Ageing Domain Levels and Changes Between Age 76 and 79 Years

Ageing domain	Baseline level association with age 76 BRS score			Change association with age 76 BRS score		
	*r*	*SE*	*p*	*r*	*SE*	*p*
General cognitive ability	.19	.043	<.001^†^	.019	.10	.86
Physical fitness	.20	.056	<.001^†^	−.26	.15	.074
Wellbeing	.60	.031	<.001^†^	—	—	—
*Note*. *SE* = standard error.						
^†^ Results significant at *p* < .05 after false discovery rate adjustment.						

## References

[c79] Aryee M. J., Jaffe A. E., Corrada-Bravo H., Ladd-Acosta C., Feinberg A. P., Hansen K. D., & Irizarry R. A. (2014). Minfi: A flexible and comprehensive bioconductor package for the analysis of infinium DNA methylation microarrays. Bioinformatics, 30, 1363–1369. 10.1093/bioinformatics/btu049 24478339 PMC4016708

[c1] Benjamini Y., & Hochberg Y. (1995). Controlling the false discovery rate: A practical and powerful approach to multiple testing. Journal of the Royal Statistical Society: Series B (Methodological), 57, 289–300. 10.1111/j.2517-6161.1995.tb02031.x

[c2] Blackburn E. H., Epel E. S., & Lin J. (2015). Human telomere biology: A contributory and interactive factor in aging, disease risks, and protection. Science, 350, 1193–1198. 10.1126/science.aab3389 26785477

[c3] Booth T., Starr J. M., & Deary I. (2013). Modeling multisystem biological risk in later life: Allostatic load in the Lothian birth cohort study 1936. American Journal of Human Biology, 25, 538–543. 10.1002/ajhb.22406 23754497

[c4] Buchman A. S., Boyle P. A., Wilson R. S., Leurgans S. E., Arnold S. E., & Bennett D. A. (2013). Neuroticism, extraversion, and motor function in community-dwelling older persons. The American Journal of Geriatric Psychiatry, 21, 145–154. 10.1016/j.jagp.2012.10.015 23343488 PMC3406259

[c5] Chmitorz A., Wenzel M., Stieglitz R. D., Kunzler A., Bagusat C., Helmreich I., . . . Tüscher O. (2018). Population-based validation of a German version of the Brief Resilience Scale. PLoS ONE, 13, e0192761 10.1371/journal.pone.0192761 29438435 PMC5811014

[c6] Connor K. M., & Davidson J. R. (2003). Development of a new resilience scale: The Connor-Davidson Resilience Scale (CD-RISC). Depression and Anxiety, 18, 76–82. 10.1002/da.10113 12964174

[c7] Cooper R., Hardy R., Aihie Sayer A., Ben-Shlomo Y., Birnie K., Cooper C., . . . the HALCyon Study Team (2011). Age and gender differences in physical capability levels from mid-life onwards: The harmonisation and meta-analysis of data from eight U.K. cohort studies. PLoS ONE, 6, e27899 10.1371/journal.pone.0027899 22114723 PMC3218057

[c8] Cosco T. D., Kaushal A., Hardy R., Richards M., Kuh D., & Stafford M. (2017). Operationalising resilience in longitudinal studies: A systematic review of methodological approaches. Journal of Epidemiology and Community Health, 71, 98–104. 10.1136/jech-2015-206980 27502781 PMC5256275

[c9] Cronbach L. J., & Meehl P. E. (1955). Construct validity in psychological tests. Psychological Bulletin, 52, 281–302. 10.1037/h0040957 13245896

[c10] Deary I. J., Gow A. J., Pattie A., & Starr J. M. (2012). Cohort profile: The Lothian Birth Cohorts of 1921 and 1936. International Journal of Epidemiology, 41, 1576–1584. 10.1093/ije/dyr197 22253310

[c11] Deary I. J., Gow A. J., Taylor M. D., Corley J., Brett C., Wilson V., . . . Starr J. M. (2007). The Lothian Birth Cohort 1936: A study to examine influences on cognitive ageing from age 11 to age 70 and beyond. BMC Geriatrics, 7, 28 10.1186/1471-2318-7-28 18053258 PMC2222601

[c12] Diener E., Emmons R. A., Larsen R. J., & Griffin S. (1985). The Satisfaction With Life Scale. Journal of Personality Assessment, 49, 71–75. 10.1207/s15327752jpa4901_13 16367493

[c13] Feder A., Nestler E. J., & Charney D. S. (2009). Psychobiology and molecular genetics of resilience. Nature Reviews Neuroscience, 10, 446–457. 10.1038/nrn2649 19455174 PMC2833107

[c14] Friborg O., Barlaug D., Martinussen M., Rosenvinge J. H., & Hjemdal O. (2005). Resilience in relation to personality and intelligence. International Journal of Methods in Psychiatric Research, 14, 29–42. 10.1002/mpr.15 16097398 PMC6878482

[c15] Friborg O., Hjemdal O., Rosenvinge J. H., & Martinussen M. (2003). A new rating scale for adult resilience: What are the central protective resources behind healthy adjustment? International Journal of Methods in Psychiatric Research, 12, 65–76. 10.1002/mpr.143 12830300 PMC6878238

[c16] General Register Office (1956). Classification of occupations. London, UK: HMSO.

[c17] Goldberg L. R. (2001). International personality item pool. Retrieved from https://ipip.ori.org/

[c18] Gow A. J., Whiteman M. C., Pattie A., & Deary I. J. (2005). Goldberg’s ‘IPIP’ big-five factor markers: Internal consistency and concurrent validation in Scotland. Personality and Individual Differences, 39, 317–329. 10.1016/j.paid.2005.01.011

[c77] Hannum G., Guinney J., Zhao L., Zhang L., Hughes G., Sadda S., . . . Deconde R. (2013). Genome-wide methylation profiles reveal quantitative views of human aging rates. Molecular cell, 49, 359–367. 10.1016/j.molcel.2012.10.016 23177740 PMC3780611

[c19] Hardy S. E., Concato J., & Gill T. M. (2004). Resilience of community-dwelling older persons. Journal of the American Geriatrics Society, 52, 257–262. 10.1111/j.1532-5415.2004.52065.x 14728637

[c20] Harris M. A., Brett C. E., Starr J. M., Deary I. J., & McIntosh A. M. (2016). Early-life predictors of resilience and related outcomes up to 66 years later in the 6-day sample of the 1947 Scottish mental survey. Social Psychiatry and Psychiatric Epidemiology, 51, 659–668. 10.1007/s00127-016-1189-4 26880008 PMC4846692

[c21] Jeste D. V., Savla G. N., Thompson W. K., Vahia I. V., Glorioso D. K., Martin A. S., . . . Depp C. A. (2013). Association between older age and more successful aging: Critical role of resilience and depression. The American Journal of Psychiatry, 170, 188–196. 10.1176/appi.ajp.2012.12030386 23223917 PMC3593664

[c22] Johnson W., Corley J., Starr J. M., & Deary I. J. (2011). Psychological and physical health at age 70 in the Lothian Birth Cohort 1936: Links with early life IQ, SES, and current cognitive function and neighborhood environment. Health Psychology, 30, 1–11. 10.1037/a0021834 21299289

[c23] Jopp D., & Rott C. (2006). Adaptation in very old age: Exploring the role of resources, beliefs, and attitudes for centenarians’ happiness. Psychology and Aging, 21, 266–280. 10.1037/0882-7974.21.2.266 16768574

[c24] Kelley T. L. (1927). Interpretation of educational measurements. New York, NY: World Book Company.

[c25] Kuwert P., Knaevelsrud C., & Pietrzak R. H. (2014). Loneliness among older veterans in the United States: Results from the National Health and Resilience in Veterans Study. The American Journal of Geriatric Psychiatry, 22, 564–569. 10.1016/j.jagp.2013.02.013 23806682

[c26] Lamond A. J., Depp C. A., Allison M., Langer R., Reichstadt J., Moore D. J., . . . Jeste D. V. (2008). Measurement and predictors of resilience among community-dwelling older women. Journal of Psychiatric Research, 43, 148–154. 10.1016/j.jpsychires.2008.03.007 18455190 PMC2613196

[c27] Lara J., Godfrey A., Evans E., Heaven B., Brown L. J., Barron E., . . . Mathers J. C. (2013). Towards measurement of the Healthy Ageing Phenotype in lifestyle-based intervention studies. Maturitas, 76, 189–199. 10.1016/j.maturitas.2013.07.007 23932426

[c28] Laukka E. J., Dykiert D., Allerhand M., Starr J. M., & Deary I. J. (2018). Effects of between-person differences and within-person changes in symptoms of anxiety and depression on older age cognitive performance. Psychological Medicine, 48, 1350–1358. 10.1017/S0033291717002896 29039283 PMC6088541

[c29] López-Otín C., Blasco M. A., Partridge L., Serrano M., & Kroemer G. (2013). The hallmarks of aging. Cell, 153, 1194–1217. 10.1016/j.cell.2013.05.039 23746838 PMC3836174

[c30] Luciano M., Gow A. J., Harris S. E., Hayward C., Allerhand M., Starr J. M., . . . Deary I. J. (2009). Cognitive ability at age 11 and 70 years, information processing speed, and APOE variation: The Lothian Birth Cohort 1936 study. Psychology and Aging, 24, 129–138. 10.1037/a0014780 19290744

[c31] MacLeod S., Musich S., Hawkins K., Alsgaard K., & Wicker E. R. (2016). The impact of resilience among older adults. Geriatric Nursing, 37, 266–272. 10.1016/j.gerinurse.2016.02.014 27055911

[c32] Marioni R. E., Harris S. E., Shah S., McRae A. F., von Zglinicki T., Martin-Ruiz C., . . . Deary I. J. (2016). The epigenetic clock and telomere length are independently associated with chronological age and mortality. International Journal of Epidemiology, 45, 424–432. 10.1093/ije/dyw041 27075770 PMC4864882

[c33] Marioni R. E., Shah S., McRae A. F., Chen B. H., Colicino E., Harris S. E., . . . Deary I. J. (2015). DNA methylation age of blood predicts all-cause mortality in later life. Genome Biology, 16, 25 10.1186/s13059-015-0584-6 25633388 PMC4350614

[c35] Martin-Ruiz C., Saretzki G., Petrie J., Ladhoff J., Jeyapalan J., Wei W., . . . von Zglinicki T. (2004). Stochastic variation in telomere shortening rate causes heterogeneity of human fibroblast replicative life span. The Journal of Biological Chemistry, 279, 17826–17833. 10.1074/jbc.M311980200 14963037

[c36] Masten A. S. (2016). Resilience in developing systems: The promise of integrated approaches. European Journal of Developmental Psychology, 13, 297–312. 10.1080/17405629.2016.1147344

[c37] Masten A. S., Hubbard J. J., Gest S. D., Tellegen A., Garmezy N., & Ramirez M. (1999). Competence in the context of adversity: Pathways to resilience and maladaptation from childhood to late adolescence. Development and Psychopathology, 11, 143–169. 10.1017/S0954579499001996 10208360

[c38] McArdle J. J. (2009). Latent variable modeling of differences and changes with longitudinal data. Annual Review of Psychology, 60, 577–605. 10.1146/annurev.psych.60.110707.163612 18817479

[c39] McEwen B. S. (1998). Stress, adaptation, and disease: Allostasis and allostatic load. Annals of the New York Academy of Sciences, 840, 33–44. 10.1111/j.1749-6632.1998.tb09546.x 9629234

[c40] Mizuno Y., Hofer A., Suzuki T., Frajo-Apor B., Wartelsteiner F., Kemmler G., . . . Uchida H. (2016). Clinical and biological correlates of resilience in patients with schizophrenia and bipolar disorder: A cross-sectional study. Schizophrenia Research, 175, 148–153. 10.1016/j.schres.2016.04.047 27185483

[c41] Muthén L. K., & Muthén B. O. (2014). Mplus user’s guide (7th ed.). Los Angeles, CA: Author.

[c42] Navrady L. B., Adams M. J., Chan S. W. Y., Ritchie S. J., McIntosh A. M., & the Major Depressive Disorder Working Group of the Psychiatric Genomics Consortium (2018). Genetic risk of major depressive disorder: The moderating and mediating effects of neuroticism and psychological resilience on clinical and self-reported depression. Psychological Medicine, 48, 1890–1899. 10.1017/S0033291717003415 29183409 PMC6088772

[c43] Neeleman J., Bijl R., & Ormel J. (2004). Neuroticism, a central link between somatic and psychiatric morbidity: Path analysis of prospective data. Psychological Medicine, 34, 521–531. 10.1017/S0033291703001193 15259837

[c44] Office of Population Censuses and Surveys (1980). Classification of occupations 1980. London, UK: HMSO.

[c45] Ong A. D., Bergeman C. S., Bisconti T. L., & Wallace K. A. (2006). Psychological resilience, positive emotions, and successful adaptation to stress in later life. Journal of Personality and Social Psychology, 91, 730–749. 10.1037/0022-3514.91.4.730 17014296

[c46] Oshio A., Taku K., Hirano M., & Saeed G. (2018). Resilience and Big Five personality traits: A meta-analysis. Personality and Individual Differences, 127, 54–60. 10.1016/j.paid.2018.01.048

[c47] Perna L., Mielck A., Lacruz M. E., Emeny R. T., Holle R., Breitfelder A., & Ladwig K. H. (2012). Socioeconomic position, resilience, and health behaviour among elderly people. International Journal of Public Health, 57, 341–349. 10.1007/s00038-011-0294-0 21912944

[c48] R Core Team (2013). R: A language and environment for statistical computing. Vienna, Austria: R Foundation for Statistical Computing Retrieved from https://www.R-project.org/

[c49] Rodríguez-Rey R., Alonso-Tapia J., & Hernansaiz-Garrido H. (2016). Reliability and validity of the Brief Resilience Scale (BRS) Spanish Version. Psychological Assessment, 28, e101–e110. 10.1037/pas0000191 26502199

[c50] Salthouse T. A. (2004). What and when of cognitive aging. Current Directions in Psychological Science, 13, 140–144. 10.1111/j.0963-7214.2004.00293.x PMC4219741 25382943

[c51] Scottish Council for Research in Education (1949). The trend of Scottish Intelligence: A comparison of the 1947 and 1932 surveys of the intelligence of eleven-year-old pupils. London, UK: University of London Press.

[c78] Shah S., McRae A. F., Marioni R. E., Harris S. E., Gibson J., Henders A. K., . . . Murphy L. (2014). Genetic and environmental exposures constrain epigenetic drift over the human life course. Genome Research, 24, 1725–1733. 10.1101/gr.176933.114 25249537 PMC4216914

[c52] Silverman M. N., & Deuster P. A. (2014). Biological mechanisms underlying the role of physical fitness in health and resilience. Interface Focus. Advance online publication 10.1098/rsfs.2014.0040 PMC4142018 25285199

[c53] Simeon D., Yehuda R., Cunill R., Knutelska M., Putnam F. W., & Smith L. M. (2007). Factors associated with resilience in healthy adults. Psychoneuroendocrinology, 32, 1149–1152. 10.1016/j.psyneuen.2007.08.005 17913377

[c54] Slavin M. J., Brodaty H., Kochan N. A., Crawford J. D., Trollor J. N., Draper B., & Sachdev P. S. (2010). Prevalence and predictors of “subjective cognitive complaints” in the Sydney Memory and Ageing Study. The American Journal of Geriatric Psychiatry, 18, 701–710. 10.1097/JGP.0b013e3181df49fb 21491631

[c55] Smith B. W., Dalen J., Wiggins K., Tooley E., Christopher P., & Bernard J. (2008). The brief resilience scale: Assessing the ability to bounce back. International Journal of Behavioral Medicine, 15, 194–200. 10.1080/10705500802222972 18696313

[c56] Smith B. W., Tooley E. M., Christopher P. J., & Kay V. S. (2010). Resilience as the ability to bounce back from stress: A neglected personal resource? The Journal of Positive Psychology, 5, 166–176. 10.1080/17439760.2010.482186

[c57] Southwick S. M., Bonanno G. A., Masten A. S., Panter-Brick C., & Yehuda R. (2014). Resilience definitions, theory, and challenges: Interdisciplinary perspectives. European Journal of Psychotraumatology. Advance online publication 10.3402/ejpt.v5.25338 PMC4185134 25317257

[c58] Stainton A., Chisholm K., Upthegrove R., & Wood S. (2018). Neuropsychological functioning as a predictor of psychological resilience: Preliminary results from the PRONIA Study. Schizophrenia Bulletin, 44, s357–s358. 10.1093/schbul/sby018.871

[c59] Steffens D. C., Fisher G. G., Langa K. M., Potter G. G., & Plassman B. L. (2009). Prevalence of depression among older Americans: The Aging, Demographics and Memory Study. International Psychogeriatrics, 21, 879–888. 10.1017/S1041610209990044 19519984 PMC2747379

[c60] Taylor A. M., Pattie A., & Deary I. J. (2018). Cohort profile update: The Lothian Birth Cohorts of 1921 and 1936. International Journal of Epidemiology, 47, 1042–1042r. 10.1093/ije/dyy022 29546429 PMC6124629

[c61] Taylor A. M., Ritchie S. J., & Deary I. J. (2017). Associations of intelligence across the life course with optimism and pessimism in older age. Intelligence, 62, 79–88. 10.1016/j.intell.2017.03.002 28626274 PMC5466381

[c62] Tennant R., Hiller L., Fishwick R., Platt S., Joseph S., Weich S., . . . Stewart-Brown S. (2007). The Warwick-Edinburgh Mental Well-Being Scale (WEMWBS): Development and UK validation. Health and Quality of Life Outcomes. Advance online publication 10.1186/1477-7525-5-63 PMC2222612 18042300

[c64] Wagnild G. M., & Young H. M. (1993). Development and psychometric evaluation of the Resilience Scale. Journal of Nursing Measurement, 1, 165–178. Retrieved from https://sapibg.org/download/1054-wagnild_1993_resilience_scale_2.pdf 7850498

[c65] Wang B., & Dong X. (2018). The association between personality and loneliness: Findings from a community-dwelling Chinese aging population. Gerontology & Geriatric Medicine, 4, 1–9. 10.1177/2333721418778181 PMC6050618 30035191

[c66] Wechsler D. (1998a). WAIS-III UK administration and scoring manual. London, UK: Psychological Corporation.

[c67] Wechsler D. (1998b). WMS-III UK administration and scoring manual. London, UK: Psychological Corporation.

[c68] Widaman K. F., Ferrer E., & Conger R. D. (2010). Factorial invariance within longitudinal structural equation models: Measuring the same construct across time. Child Development Perspectives, 4, 10–18. 10.1111/j.1750-8606.2009.00110.x 20369028 PMC2848495

[c69] Wild K., Wiles J. L., & Allen R. E. (2013). Resilience: Thoughts on the value of the concept for critical gerontology. Ageing & Society, 33, 137–158. 10.1017/S0144686X11001073

[c70] Wilson R. S., Bennett D. A., Mendes de Leon C. F., Bienias J. L., Morris M. C., & Evans D. A. (2005). Distress proneness and cognitive decline in a population of older persons. Psychoneuroendocrinology, 30, 11–17. 10.1016/j.psyneuen.2004.04.005 15358438

[c71] Wilson R. S., Krueger K. R., Gu L., Bienias J. L., Mendes de Leon C. F., & Evans D. A. (2005). Neuroticism, extraversion, and mortality in a defined population of older persons. Psychosomatic Medicine, 67, 841–845. 10.1097/01.psy.0000190615.20656.83 16314587

[c72] Windle G. (2011). What is resilience? A review and concept analysis. Reviews in Clinical Gerontology, 21, 152–169. 10.1017/S0959259810000420

[c73] Yang Y., & Wen M. (2014). Psychological resilience and the onset of activity of daily living disability among older adults in China: A nationwide longitudinal analysis. The Journals of Gerontology: Series B, Psychological Sciences and Social Sciences, 70, 470–480. 10.1093/geronb/gbu068 24898031

[c74] Yi J. P., Vitaliano P. P., Smith R. E., Yi J. C., & Weinger K. (2008). The role of resilience on psychological adjustment and physical health in patients with diabetes. British Journal of Health Psychology, 13, 311–325. 10.1348/135910707X186994 17535497 PMC2899486

[c75] Zeng Y., & Shen K. (2010). Resilience significantly contributes to exceptional longevity. Current Gerontology and Geriatrics Research. Advance online publication 10.1155/2010/525693 PMC3004383 21197075

[c76] Zigmond A. S., & Snaith R. P. (1983). The hospital anxiety and depression scale. Acta Psychiatrica Scandinavica, 67, 361–370. 10.1111/j.1600-0447.1983.tb09716.x 6880820

