# Correction to Taylor et al. (2019)

**DOI:** 10.1037/pag0000462

**Published:** 2020-03-09

The article “Associations Between Brief Resilience Scale Scores and Ageing-Related Domains in the Lothian Birth Cohort 1936,” by Adele M. Taylor, Stuart J. Ritchie, Ciara Madden, and Ian J. Deary (*Psychology and Aging*, advance online publication, November 4, 2019, http://dx.doi.org/10.1037/pag0000419), should have been published under the terms of the Creative Commons Attribution License (CC BY 3.0). Therefore, the article was amended to list the authors as copyright holders, and information about the terms of the CC BY 3.0 was added to the author note. In addition, the article is now open access. All versions of this article have been corrected.

