# Supplementary material for: Associations Between Brief Resilience Scale Scores and Ageing-Related Domains in the Lothian Birth Cohort 1936
Source: Psychol Aging. 2019 Nov 4;35(3):329–44. doi: 10.1037/pag0000419 (PMC7161361; doi:10.1037/pag0000419)
Supplement: Supplementary file 1 [file PAG-2019-0483_Suppl.docx]

Supplementary Table 1. Correlations between resilience and concurrently measured key ageing domain variables at age 76.

|  | 1 | 2 | 3 | 4 | 5 | 6 | 7 | 8 | 9 | 10 | 11 | 12 | 13 | 14 | 15 | 16 |
| --- | --- | --- | --- | --- | --- | --- | --- | --- | --- | --- | --- | --- | --- | --- | --- | --- |
| 1. Resilience |  |  |  |  |  |  |  |  |  |  |  |  |  |  |  |  |
| 1. Symbol search | .16** | - |  |  |  |  |  |  |  |  |  |  |  |  |  |  |
| 1. Digit symbol substitution | .15** | .64** | - |  |  |  |  |  |  |  |  |  |  |  |  |  |
| 1. Matrix reasoning | .16** | .42** | .37** | - |  |  |  |  |  |  |  |  |  |  |  |  |
| 1. Letter-number sequencing | .090* | .37** | .40** | .35** | - |  |  |  |  |  |  |  |  |  |  |  |
| 1. Digit span backwards | .082* | .33** | .31** | .37** | .56** | - |  |  |  |  |  |  |  |  |  |  |
| 1. Block design | .091* | .51** | .41** | .56** | .30** | .29** | - |  |  |  |  |  |  |  |  |  |
| 1. Grip strength | .13** | .15** | .14** | .15** | .090* | .11** | .16** | - |  |  |  |  |  |  |  |  |
| 1. Lung function | .031 | .14** | .13** | .09* | <.001 | -.046 | .13** | .21** | - |  |  |  |  |  |  |  |
| 1. Walk speed | -.11** | -.30** | -.32** | -.20** | -.18** | -.17** | -.18** | -.27** | -.22** | - |  |  |  |  |  |  |
| 1. Allostatic load | -.003 | -.034 | -.073 | -.11** | -.088* | -.12** | -.039 | -.041 | -.080* | .12** | - |  |  |  |  |  |
| 1. Telomere length | -.001 | -.029 | .007 | .006 | .011 | -.025 | -.030 | -.077* | -.002 | .04 | .012 | - |  |  |  |  |
| 1. Methylation age acceleration | .027 | -.065 | -.11** | -.016 | .007 | .054 | -.011 | .007 | -.047 | .03 | .087 | -.14** | - |  |  |  |
| 1. WEMWBS | .47** | .19** | .20** | .20** | .13** | .12** | .15** | .15** | .10* | -.26** | -.013 | -.018 | -.066 | - |  |  |
| 1. SWLS | .30** | .11** | .12** | .080* | .053 | .064 | .11** | .051 | .091* | -.18** | -.024 | -.003 | -.004 | .53** | - |  |
| 1. HADS | -.46** | -.22** | -.24** | -.24** | -.19** | -.15** | -.26** | -.17** | -.10** | .19** | .078* | -.073 | .034 | -.53** | -.39** | - |

Grip strength, best of three attempts from right hand (kg); Lung function, forced expiratory volume in 1 second; Walk speed, time to walk 6m in seconds WEMWBS, Warwick-Edinburgh Mental Wellbeing Scale, SWLS, Satisfaction with Life Scale; HADS, Hospital Anxiety and Depression Scale. *significant at *p*-value <0.05; ** significant at *p*-value <0.01

Supplementary Table 2. Correlations between resilience at age 76 and key ageing domain variables measured at age 79

| Measures | 1 | 2 | 3 | 4 | 5 | 6 | 7 | 8 | 9 | 10 | 11 | 12 | 13 | 14 | 15 | 16 |
| --- | --- | --- | --- | --- | --- | --- | --- | --- | --- | --- | --- | --- | --- | --- | --- | --- |
| 1. Resilience |  |  |  |  |  |  |  |  |  |  |  |  |  |  |  |  |
| 1. Symbol search | .057 | - |  |  |  |  |  |  |  |  |  |  |  |  |  |  |
| 1. Digit symbol substitution | .11* | .62** | - |  |  |  |  |  |  |  |  |  |  |  |  |  |
| 1. Matrix reasoning | .12** | .44** | .36** | - |  |  |  |  |  |  |  |  |  |  |  |  |
| 1. Letter-number sequencing | .073 | .44** | .40** | .36** | - |  |  |  |  |  |  |  |  |  |  |  |
| 1. Digit span backwards | .11* | .36** | .38** | .35** | .57** | - |  |  |  |  |  |  |  |  |  |  |
| 1. Block design | .12** | .50** | .38** | .54** | .34** | .28** | - |  |  |  |  |  |  |  |  |  |
| 1. Grip strength | .086 | .13** | .093* | .084 | .031 | .005 | .18** | - |  |  |  |  |  |  |  |  |
| 1. Lung function | -.020 | .10* | .11* | .041 | -.040 | .029 | .12** | .24** | - |  |  |  |  |  |  |  |
| 1. Walk speed | -.060 | -.25** | -.34** | -.23** | -.15** | -.13** | -.25** | -.27** | -.28** | - |  |  |  |  |  |  |
| 1. Allostatic load | -.007 | -.066 | -.031 | -.086* | -.12** | -.11** | -.056 | .001 | -.11* | .17** | - |  |  |  |  |  |
| 1. Telomere length | 002 | -.012 | .032 | -.038 | -.038 | -.068 | -.010 | -.069 | -.055 | -.018 | .021 | - |  |  |  |  |
| 1. Methylation age acceleration | .065 | -.057 | -.11* | -.006 | .012 | -.020 | .055 | -.043 | -.059 | .068 | .10* | -.041 | - |  |  |  |
| 1. WEMWBS | .44** | .14** | .13** | .11* | .040 | .038 | .12** | .16** | .040 | -.16** | .027 | .015 | -.079 | - |  |  |
| 1. SWLS | .39** | .089* | .093* | .065 | .045 | .076 | .12** | .12** | .045 | -.11* | -.10* | .034 | .014 | .56** | - |  |
| 1. HADS | -.46** | -.15** | -.18** | -.12** | -.038 | -.081 | -.17** | -.13** | -.058 | .20** | .048 | -.023 | -.018 | -.54** | -.44** | - |

Grip strength, best of three attempts from right hand (kg); Lung function, forced expiratory volume in 1 second; Walk speed, time to walk 6m in seconds WEMWBS, Warwick-Edinburgh Mental Wellbeing Scale, SWLS, Satisfaction with Life Scale; HADS, Hospital Anxiety and Depression Scale. *significant at *p*-value <0.05; ** significant at *p*-value <0.01
